# Determination of split renal function in voluntary renal donors by multidetector computed tomography and nuclear renography: How well do they correlate?

**DOI:** 10.4102/sajr.v25i1.2009

**Published:** 2021-03-05

**Authors:** Hira Lal, Anuradha Singh, Raghunandan Prasad, Priyank Yadav, Javed Akhtar, Sukanta Barai, Prabhakar Mishra, Dharmendra Bhadauria, Anupma Kaul, Narayan Prasad, Pragati Verma

**Affiliations:** 1 Department of Radiology, Faculty of Health Sciences, Sanjay Gandhi Post Graduate Institute of Medical Sciences, Lucknow, India; 2 Department of Urology and Renal Transplantation, Faculty Health Sciences, Sanjay Gandhi Post Graduate Institute of Medical Sciences, Lucknow, India

**Keywords:** nuclear renography, split renal function, computed tomography, volumetry, attenuation

## Abstract

**Background:**

The use of computed tomography (CT) for estimation of split renal function (SRF) has been reported previously. However, most of these studies have small samples, and many do not account for the renal attenuation at CT.

**Objective:**

The aim of this study was to compare multidetector computed tomography (MDCT) volumetry-attenuation-based SRF with that obtained via Tc99m-diethylenetriaminepentaacetic acid (DTPA) renal scintigraphy in voluntary renal donors.

**Methods:**

Between January 2017 and January 2020, 526 voluntary renal donors were enrolled prospectively. All donors underwent contrast CT and DTPA scan before surgery. The semiautomatic region of interest (ROI) tool was applied slice by slice on axial CT images acquired in the arterial phase. The renal contour was drawn semiautomatically with mouse clicks around the renal parenchyma, and the renal volume was ascertained. Using renal volume and attenuation, SRF was determined and compared with results obtained at DTPA imaging.

**Results:**

The mean age was 44.91 ± 10.97 years (mean ± s.d.). There was no significant difference in SRF based on DTPA and MDCT volumetry for the left kidney (49.18% ± 3.40% vs. 49.15% ± 3.38%, *p* = 0.540) and for the right kidney (50.82% ± 3.40% vs. 50.86% ± 3.39%, *p* = 0.358). A very good correlation was observed between the two methods for the left kidney (*r* = 0.953, *p* = 0.000) and the right kidney (*r* = 0.955, *p* = 0.000). On simple linear regression analysis, 90.8% of DTPA SRF values for the left kidney and 91.3% of DTPA SRF values for the right kidney could be predicted correctly using the corresponding MDCT SRF values.

**Conclusion:**

MDCT volumetry-attenuation-derived estimation of SRF for living renal donors could be an alternative to renal scintigraphy-based SRF estimation.

## Introduction

The incidence of end-stage renal disease is increasing steadily, resulting in an escalated number of renal transplantations worldwide in recent years.^1^ Live renal donors are the most common source of renal allografts even now, and they typically undergo extensive preoperative evaluation to gain information about their renal vascular anatomy, as well as function. Renal scintigraphy is typically used for assessment of split renal function (SRF), and contrast-enhanced computed tomography (CT) is used for vasculature and anatomical details. However, renal scintigraphy is not absolutely error free, and the results differ with changes in distance of the kidney from the gamma camera (such as in obese patients).^2^ Furthermore, there exists considerable interobserver and intraobserver variability in nuclear scintigraphy reports, which can be estimated up to 8%.^3^ The renal excretion of iodinated contrast materials used for contrast-enhanced CT occurs in a manner similar to the reference for estimation of glomerular filtration rate (GFR) – inulin; calculation of clearance of such contrast provides an accurate measurement of the GFR. A comparison of contrast accumulation and/or excretion by the kidneys can thus accurately provide the SRF of individual kidneys.

The concept of CT-based estimation of SRF evolved more than two decades ago when Dawson and Peter based their proposition on the plasma clearance of contrast according to the Rutland-Patlak plot.^4^ Recent studies in living kidney donors (LKD) have shown that SRF can be evaluated preoperatively by CT-based analysis of the kidney volume.^5,6^ In these studies, SRF was calculated by CT and compared with the Technetium-99m – diethylenetriaminepentaacetic acid (DTPA)-SRF. Multidetector computed tomography (MDCT) accurately determines the relative contribution of each kidney to overall kidney function, which is of critical importance in the preoperative evaluation of a LKD. If a disparity in kidney function exists, the donor should be left with the better functioning kidney to optimise donor safety.^7^

Several authors have reported the use of CT for estimation of SRF, using different principles and algorithms; however, most of them are unpopular owing to complicated protocols, inaccurate attenuation correction or multiple assumptions and interpolations. The aim of this study was to compare CT volumetry-attenuation based SRF with that obtained at Tc99m-DTPA renal scintigraphy using a simplified standardised protocol.

## Methodology

This study was a hospital-based analytical study conducted at a tertiary care hospital in Northern India from 01 January 2017 to 31 January 2020. All prospective renal donors who were able to follow breathing and scanning instructions were included in the study.

Computed tomography volumetry was performed using post-processing software. Additionally, each donor underwent a Technetium-99m DTPA renal scan before surgery. Considering a 5% margin of error and a confidence interval of 98%, for a modest 40% acceptance rate (considering the standard discard rate of > 50% for expanded donor criteria),^8^ the estimated sample size was 522.

Computed tomography imaging was acquired with a 64-channel multidetector scanner (Brilliance, Philips Medical System, Eindhoven, The Netherlands) at our institution. The study was performed using a split bolus of contrast for combined arterial and nephrographic phase imaging followed by CT urography. Each donor was required to drink 1000 mL of plain water, 45 min prior to the examination. Unenhanced CT acquisition was extended from above the kidney to the pubic symphysis with breath-hold in inspiration. The enhanced CT acquisition included the area from above the diaphragm to below the pubic symphysis with breath-hold in inspiration. About 40 mL of non-ionic contrast medium (Visipaque, GE Healthcare, 320 mg/mL) was administered intravenously using an automatic injector (STELLANT-MEDRAD Version 102.OSH) at a rate of 2 mL/s through an 18 G canula placed in the antecubital fossa. After a 25 s delay, another 60 mL of contrast medium was injected at a rate of 4 mL/s, followed by 30 mL of normal saline at 3.5 mL/s. The region of interest (ROI) was kept outside the body to avoid misinterpretation of the ROI with high-density structures (vertebrae) adjacent to aorta because of patient movements; the monitoring scan started 5 s after the second phase of contrast injection. Subsequently, image acquisition was commenced manually, when high-density contrast was seen in the abdominal aorta at the level of diaphragm. After 7 min – 10 min, another acquisition was performed for the excretory phase extending from above the kidney to the pubic symphysis with breath-hold in inspiration.

Radiation dose was recorded for each patient. In order to further reduce the radiation dose, tube current was reduced from 250 mAs to 150 mAs, and slice thickness was kept at 3.0 mm on unenhanced and excretory phase acquisitions without affecting the image quality. Slice thickness was 0.9 mm on the combined arterial-venous phase. The acquisition times for the unenhanced phase, combined arterial-venous phase and the excretory phase were 5 s – 7 s, 6 s – 8 s and 5 s – 7 s, respectively.

### Split renal function

**FIGURE 1:**
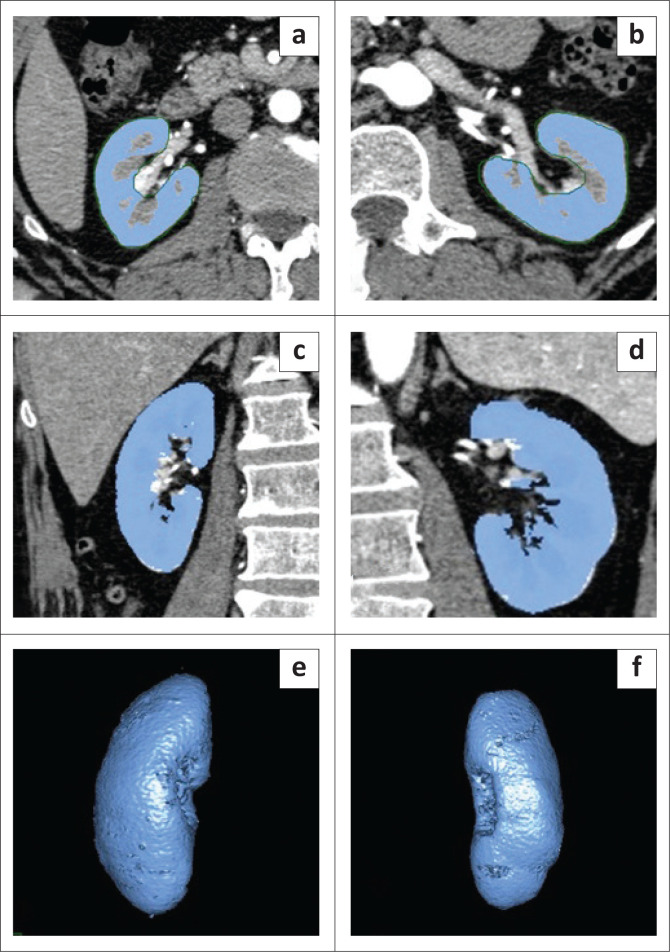
The process of measuring kidney volume. The semiautomatic region of interest tool was applied slice by slice on axial arterial phase images (a) and (b), as well as on coronal arterial phase images (c) and (d). The semiautomated software estimated the kidney volume (e) and (f).

The semiautomatic ROI tool was applied slice by slice in the axial plane on arterial phase images. The renal contour was drawn semiautomatically with mouse clicks around the renal parenchyma (Figure 1a–d), and the software estimated the renal volume (Figure 1e–f). The collecting system, fat in the renal sinus and renal space occupying lesions of fluid density (cysts) were excluded by pre-set software thresholds. Pelvicalyceal and perirenal fat, cysts and hydronephrotic pelvicalyceal fluid were excluded by applying a lower window limit of 40 HU. Any calculi and opacified intrarenal collecting systems were excluded by applying an upper window limit of 350 HU. The pre-set soft-tissue window (window width, 350 HU; window level, 40 HU) could be changed at will. Finally, the software calculated the renal volume (V) for each kidney separately.

The mean attenuation of the kidney was recorded on the unenhanced images and subtracted from the mean attenuation taken at the same level on combined arterial and nephrographic phase images to attain the enhancing attenuation of the kidney (A). The total attenuation value contributed by the contrast medium in each kidney was calculated and assumed to be proportional to that kidney’s relative function. Right kidney SRF was then calculated as the product of volume and attenuation for the right kidney divided by the sum of the product of volume and attenuation for both kidneys. SRF_R_ = (RV × RA)/ (RV × RA) + (LV × LA).^9^ Similarly, SRF_L_ was also calculated.

### Estimaton of split renal function by renal scintigraphy

For differential renal function, an angiographic perfusion study was performed using 1 mCi of Tc99m – DTPA. After ensuring adequate hydration and voiding before the commencement of the study, the donors were placed in a supine position, and the scintillation camera detector was positioned so that the bifurcation of the aorta, iliac arteries, urinary bladder and kidneys appeared in the camera field. Three-second sequential exposures were obtained, whilst activity was clearly localised in the arterial system and kidney. This was followed by a 40-s static image to evaluate renal size and shape. Subsequently, activity was quantified over the individual kidneys and bladder using either the split crystal or the ROI mode. The static DTPA scintigrams were used to compare the size and shape of the kidney between studies, as well as to assess the tissue to background ratio, which decreased with the deterioration of renal function.^10^ The ROIs plotted around each kidney were used to generate renograms after subtracting the area-normalised background ROIs. The uptake part of the renogram was used to calculate the SRF.

### Statistical analysis

Normality of continuous variables was assessed, and variables were normally distributed when the standard normal variate (*Z*) value of the skewness was ±3.29. Continuous variables were presented as mean ± standard deviation (s.d.), median (inter-quartile range) and range (minimum-maximum), whilst categorical variables were presented as frequency (percentage). The paired sample *t*-test was used to test the change in the mean score between paired observations (pre-post). For comparison of the means between two unpaired groups, the independent sample *t*-test was used, whilst to compare the means for more than two groups, the one-way analysis of variance (ANOVA) test was used, followed by multiple comparisons using the Bonferroni method. In order to compare the proportions between the groups, the Chi-square test was used, and to assess the linear relationship between two continuous variables, the Pearson correlation coefficient was used. Similarly, to predict one continuous variable from another continuous variable, simple (univariate) linear regression analysis was utilised. A scatter diagram was used to test the linear relationship between the two continuous variables and the Bland Altman plot to test the linear relationship between one variable (DTPA) and the difference between two variables (DTPA – MDCT). A *p-*value < 0.05 was considered as statistically significant. The statistical package for social sciences, version 23 (SPSS-23, IBM, Chicago, USA) was used for data analysis.

## Ethical consideration

The study was approved by the institutional ethics committee of Sanjay Gandhi Post Graduate Institute of Medical Sciences (IEC code: 2017-94-MD-EXP), and the procedures followed were in accordance with the Helsinki Declaration of 1975, as revised in 2000.^11^ After obtaining proper informed consent, multiphasic CT was performed in each case.

## Results

During the study period, 550 kidney donors were enrolled. Of these donors, 24 were excluded from the study as they were unable to adequately hold their breath during the CT scanning, leading to motion artefacts. The mean age of the remaining 526 donors was 44.91 ± 10.97 years (mean ± s.d.) and the age range was 18–76 years. The majority of the participating donors were women (*n* = 429, 81.6%).

### Renal volume measurements using MDCT

The mean volumes of the left and right kidneys were 112.65 cc ± 16.08 cc and 114.74 cc ± 16.14 cc, respectively. There was a significantly higher volume in men when compared with women for the left and right kidneys (Table 1). The mean volumes of the left and right kidneys were also assessed per age group of the kidney donors. There was a linear trend (negative correlation) in the volume and age, indicating a decrease in volume with increasing age for the left kidney (*r* = −0.219, *p* = 0.000) and for the right kidney (*r* = −0.178, *p* = 0.000).

**TABLE 1:** Kidney volume and gender.

Volume(cc)	Variable	Male (*n* = 97)	Female (*n* = 429)	Total (*N* = 526)	*p*
Left kidney	Mean ± s.d.	126.03 ± 13.99	109.63 ± 14.96	112.65 ± 16.08	< 0.001
	Median	127	109	113	-
	IQR	118–135	98–121	100–124	-
Right kidney	Mean ± s.d.	127.03 ± 15.46	111.97 ± 14.96	114.74 ± 16.14	< 0.001
	Median	127	112	115	-
	IQR	119–138	100–123	102–126	-

Note: Independent sample *t*-test used; *p* < 0.05 is considered statistically significant.

s.d., standard deviation.

### Split renal function measurement using computed tomography volumetry and DTPA

The mean (± s.d.) CT-derived SRF for left renal donors was 49.15% ± 3.38% (range: 35.8% – 58%), whilst that for right renal donors was 50.86% ± 3.39% (range: 41.2% – 64.2%). The mean (±s.d.) DTPA-derived SRF for left renal donors was 49.18% ± 3.40% (range: 37% – 60%), whilst that for right renal donors was 50.82% ± 3.40% (range: 40% – 63%). When we analysed the measurements between the two methods, there was no significant difference in SRF based on DTPA and MDCT volumetry for the left kidney (49.18% ± 3.40% vs. 49.15% ± 3.38%, *p* = 0.540) nor for the right kidney (50.82% ± 3.40% vs. 50.86% ± 3.39%, *p* = 0.358; Table 2).

**TABLE 2:** Split renal function (%) measurements with multidetector computed tomography and diethylenetriaminepentaacetic acid.

Kidney	DTPA		MDCT		*r*	*p*	Paired differences	
	Mean ± s.d.	Range	Mean ± s.d.	Range			Mean ± s.d.	95% CI of mean
Left (*n* = 526)	49.18 ± 3.40	37–60	49.15 ± 3.38	35.8–58.8	0.953	< 0.001	0.03 ± 1.04	−0.06 to 0.12
Right (*n* = 526)	50.82 ± 3.40	40–63	50.86 ± 3.39	41.2–64.2	0.955	< 0.001	−0.04 ± 1.02	−0.13 to 0.05

Note: Paired sample *t*-test used; *r*, Pearson’s correlation coefficient; *p* < 0.05 is considered statistically significant; Left kidney, *p* = 0.540; Right kidney, *p* = 0.358.

DTPA, diethylenetriaminepentaacetic acid; MDCT, multidetector computed tomography; CI, confidence interval; s.d., standard deviation.

**FIGURE 2:**
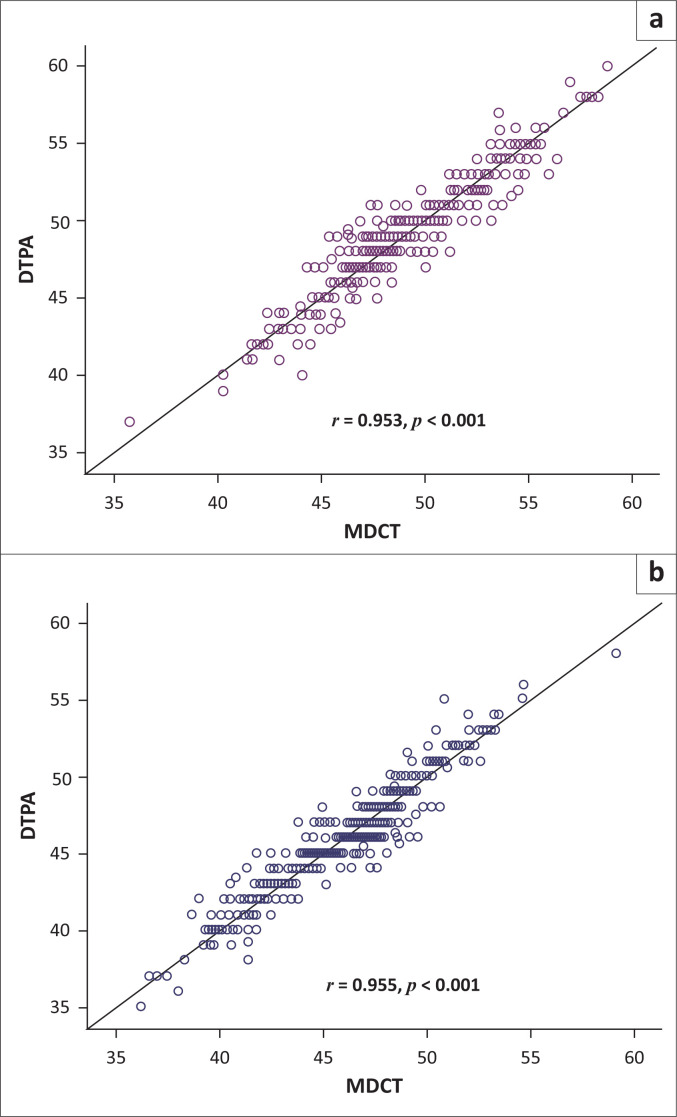
Scatter diagram showing a strong linear relationship between diethylenetriaminepentaacetic acid and multidetector computed tomography measurements: (a) left kidney and (b) right kidney.

**FIGURE 3:**
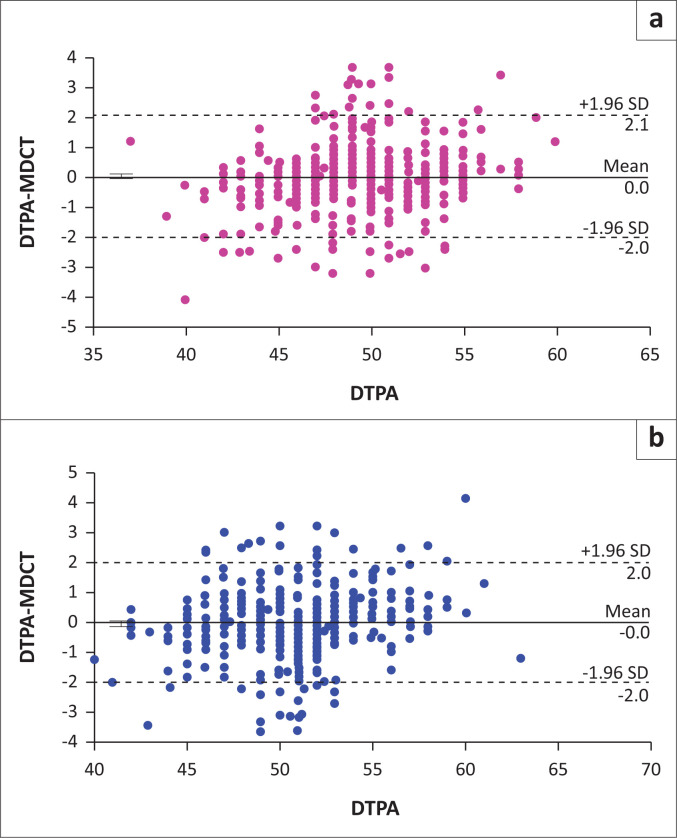
The Bland Altman plot showing a strong linear relationship between diethylenetriaminepentaacetic acid and multidetector computed tomography measurements for (a) the left kidney and (b) the right kidney.

**FIGURE 4:**
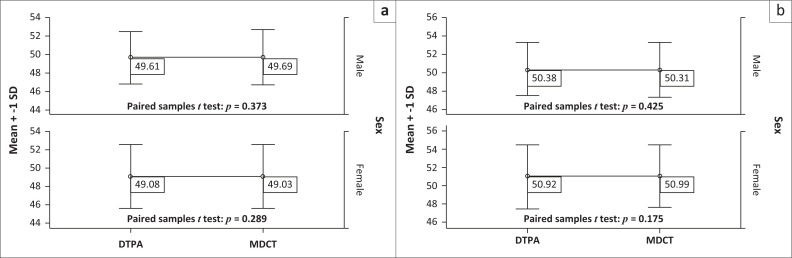
An error bar graph depicting the comparison of split renal function between diethylenetriaminepentaacetic acid and multidetector computed tomography for male and female in (a) the left kidney and (b) the right kidney.

The Pearson correlation coefficient was calculated for the SRF measurements between the two methods. The results revealed that there was a very good correlation between the two methods for the left kidney (*r* = 0.953, *p* = 0.000) and the right kidney (*r* = 0.955, *p* = 0.000; Table 2, Figure 2). The mean difference and 95% confidence intervals (CIs) were calculated using the paired sample *t*-test (Table 2); 95% CI of the absolute limits of agreement between DTPA and difference between the two methods (DTPA and MDCT) was calculated using the Bland Altman plot for left kidney (Figure 3a) and right kidney (Figure 3b) individually. Figure 3 shows that most of the values of DTPA and MDCT differences were within ± 1.96 s.d. and only a few values of differences were outside or above ± 1.96 s.d. No significant differences were observed between men and women in DTPA-derived SRF and MDCT-derived SRF for the left and right kidneys individually (Table 3, Figure 4).

**TABLE 3:** Comparison of split renal function between diethylenetriaminepentaacetic acid and multidetector computed tomography.

Variable	Sex	*N*	Mean	s.d.	*p*
DTPA_LEFT_SRF %	-	-	-	-	0.171
	Male	97	49.61	2.88	-
	Female	429	49.08	3.50	-
DTPA_RIGHT_SRF %	-	-	-	-	0.160
	Male	97	50.38	2.89	-
	Female	429	50.92	3.51	-
MDCT_LEFT_SRF %	-	-	-	-	0.080
	Male	97	49.69	3.04	-
	Female	429	49.03	3.45	-
MDCT_RIGHT_SRF %	-	-	-	-	0.073
	Male	97	50.31	3.04	-
	Female	429	50.99	3.46	-

Note: Independent sample *t*-test used; *p* < 0.05 is considered statistically significant.

DTPA, diethylenetriaminepentaacetic acid; MDCT, multidetector computed tomography; SRF, split renal function; s.d., standard deviation.

### Prediction of DTPA derived split renal function value using MDCT volumetry-based split renal function

**FIGURE 5:**
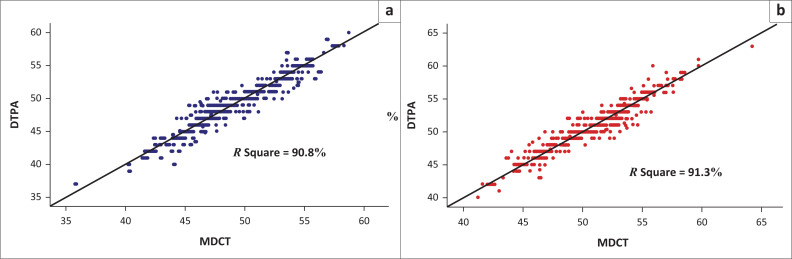
Scatter diagram showing multidetector computed tomography prediction of the diethylenetriaminepentaacetic acid split renal function (outcome) by linear regression analysis for (a) the left kidney and (b) the right kidney.

In order to predict the DTPA SRF using MDCT SRF, simple linear regression analysis was used. For the left kidney, 90.8% (Table 4, Figure 5a) and for the right kidney, 91.3% (Table 4, Figure 5b) of the DTPA SRF could be predicted correctly using the corresponding MDCT SRF values.

**TAble 4:** Projection of nuclear renography (DTPA) split renal function (SRF) using radiological split renal function (MDCT).

Dependent variable	*R*	*R* square	Regression coefficient (*β*)	Constant (C)
Nuclear medicine SRF (Left Kidney)	0.953	0.908	0.958*	2.08*
Nuclear medicine SRF (Right Kidney)	0.955	0.913	0.958*	2.11*

Note: Simple linear regression analysis was used; *p* < 0.05 is considered statistically significant; Regression model: *Y = βX + C*; Dependent variable (*Y*): Nuclear medicine SRF; Independent variable (*X*): Radiological SRF.

## Discussion

In this study, most of the donors belonged to the 40–49 year age group (*n* = 172, 32.7%), and the majority of them included women (*n* = 429, 81.6%). In the study by Deiz et al.^12^, the mean age was 39.5 ± 11.0 years and over half of the donors in their group were women. In a similar study by Barbas et al.,^7^ the average age was 49.8 years, and 64.8% of patients were women.

The left kidney was donated in 78.4% of cases. The mean volumes of the left and right kidneys were 112.65 ± 16.08 and 114.74 ± 16.14, respectively. A significantly higher renal volume was observed in the men compared with women for the left kidney (126.03 ± 13.99 vs. 109.63 ± 14.96, *p* = 0.000) as well as the right kidney (127.03 ± 15.46 vs. 111.97 ± 14.96, *p* = 0.000). Similar differences in kidney volume amongst men and women were found in the study by Poggio et al.^13^ who also reported similar age-related changes in renal volumes.

This study attempted to present a method for CT-based estimation of SRF in renal donors using a method that is easy to reproduce and accurate for its intended purpose. A closer look at the different measurement methods used in previous studies formed the basis of the current methodology used. These can be broadly categorised into simple volumetry-based and volumetry-attenuation-based methods.

### Simple volumetry-based methods

One of the simplest methods used to calculate renal volume is the ellipsoid method. However, the limitations of such a simplified approach to renal volumetry were highlighted by Breau et al.^14^ who compared the ellipsoid method with specialised volumetric software. In their study, just over half of the volumes estimated with the ellipsoid formula were within 10% of the 3D software measured volume. In a later study, Zakhari et al.^15^ proposed that the usual correction factor underestimated the renal volume.

Most authors, subsequently, shifted to other techniques for estimation of renal volume. Diez et al.^12^ carried out a retrospective review of renal donors to assess the utility of CT for determining the SRF. Amongst the 65 donors who underwent both CT and nuclear renography, the mean difference between CT and nuclear scan SRF was 0.65 ± 3.46. They reported a significant correlation between the two modalities (*r* = 0.59; *p* = 0.000). Yanishi et al.^16^ also estimated CT-based SRF by calculating split renal volume without considering renal attenuation before and after contrast administration in 35 live renal donors. Compared with mercaptoacetyltriglycine (MAG3)-based SRF, CT renal volume-based SRF was found to correlate strongly (*r* = 0.714). Mitsui et al.^5^ in a later study found a strong correlation between both types of volumes and MAG3-measured SRF (*r* = 0.921 for renal cortical volume; *r* = 0.942 for renal parenchymal volume) using an automated volume analyser software for CT renal volumetry (SYNAPSE VINCENT version 4, FUJIFILM, Tokyo, Japan).

Despite the strong correlation demonstrated in the studies mentioned earlier, there remains a lack of consensus on the ideal volumetry method. To answer this problem, Wahba et al.^6^ compared three CT volumetry techniques (modified ellipsoid volume [MELV], smart ROI volume and renal cortex volume [RCV]) in 101 LKD for calculating the SRF. After comparing the results with MAG3 scans, they concluded that although the highest level of agreement in the study was for ROI, the RCV had a low deviation and was adjudged the most accurate technique for pre-donation SRF. Modified ellipsoid volume was also shown to have a high level of agreement with MAG3 by Soga et al.^17^ Similarly, Gardan et al.^18^ reported that whilst total kidney volume correlates well with pre-donation global GFR and post-donation GFR (*p* = 0.000), renal cortical volume has a stronger correlation for estimating pre-donation SRF and post-donation renal outcome at 1 year. Recently, Siedek et al.^19^ compared CT- and MRI-based SRF using RCV and MELV and found that RCV is more accurate and reliable than MELV for CT as well as MRI-based SRF. Similarly, Nakamura et al.^20^ reported equivalence of CT volumetry to DTPA for preoperative SRF in 34 renal donors.

In this study, using the semiautomatic ROI tool for determination of the renal volume helped us to decrease the time required for completing the volumetry for both kidneys. However, volumetry was only one part of SRF estimation, the other being measurement of attenuation.

From the study by Yanishi et al.^16^, the mean (±s.d.) volumes of the right and left kidneys were found to be 138.8 mL ± 29.4 mL and 136.1 mL ± 29.2 mL, respectively. Wahba et al.^6^ had reported in their study the mean (±s.d.) volume of the preserved kidney as 148.0 cm^3^ ± 29.1 cm^3^ (ROI) and the donated kidney as 149.5 cm^3^ ± 30.8 cm^3^ (ROI), respectively. Mitsui et al.^5^ used the total renal parenchymal volume and preserved parenchymal volume in place of separate left and right kidney volumes. The median total parenchymal volume was 278.4 mL, and the preserved parenchymal volume was 131.8 mL. This study is comparable with previous studies with regards to CT volumetry parameters.

### Volumetry-attenuation-based methods

Volumetry-based SRF estimation considers only the anatomical appearance of the kidneys without any functional implications. In patients with renal parenchymal disease, the change in volume occurs later in the disease and functional deterioration occurs earlier. Contrast uptake by the kidney is a measure of its function, and consequently, attenuation on CT after administration of contrast may be a better indicator of renal function.

The use of attenuation along with renal volume stems from the modification of the initial description by Frennby et al.^21^ El-Diasty et al.^22^ compared the GFR calculated from the CT scan with that from the MAG3 scan of 80 renal donors and proved that the two correlated (*r* = 0.54, *p* < 0.001), and that selective GFR calculation of each kidney was possible with CT alone. Their calculation of CT GFR took into account the mean attenuation value (MAV) of each kidney as well. Around the same period, Nilsson et al.^2^ retrospectively reviewed 27 renal donors, calculated the renal volume as well as the MAV using the slice summation method, and compared CT and MAG3 based on SRF. In their study, the total function of the kidney correlated very well with the volume (*r* = 0.90) and the SRF calculated from CT scan, using the difference in attenuation between excretory phase and pre-contrast scans, correlated closely with the SRF from the MAG3 scans.

Knox et al.^9^ compared MDCT-derived SRF with dimercapto succinic acid (DMSA)-derived SRF in 27 donors who underwent nephrectomy. Renal volume was calculated using Voxar 3D imaging software (version 6.3, Toshiba Medical Visualization Systems, UK), and SRF was estimated in a manner similar to that used in this study. The mean difference between MDCT-derived DRF and DMSA was 0.8% (95% CI 0.1–1.6) as reported by them. Summerlin et al.^3^ compared CT-based SRF and radionuclide-based SRF in 152 renal donors. Computed tomography-based SRF was calculated using four methods: arterial phase contrast accumulation, excretory phase contrast accumulation, simple volumetry and the Patlak method. Amongst these, it was found that excretory phase and renal volume-based SRF were most accurate and correlated well with radionuclide renography (*r* = 0.58 and 0.63, respectively). Patankar et al.^23^ used the same calculations as Summerlin et al.^3^ used for the arterial phase and excretory phase for estimation of SRF. The mean SRF of the right kidney was 50.2 ± 3.3 (range, 44.1% – 54.0%) based on the nuclear scan and was 49.0 ± 2.9 (range, 46.4% – 52.3%) based on CT with a moderate correlation between the two (*r* = 0.46). The accuracy of volume-attenuation-based SRF on CT is high, and this allows for reduction of the radiation exposure that is otherwise associated with the CT protocol for the Patlak method.^24^

In this study, there was no significant difference in SRF between Tc99m DTPA and the MDCT method for the left kidney (mean ± s.d.: 49.18 ± 3.40 vs. 49.15 ± 3.38, *p* = 0.540) as well as for the right kidney (50.82 ± 3.40 vs. 50.86 ± 3.39, *p* = 0.358). We preferred to use volumetry-attenuation-based SRF on CT rather than volumetry alone based on the premise mentioned earlier. Pearson’s correlation coefficient was also calculated for SRF measurements between the two methods. The results revealed that there was a very good correlation between the two methods for the left kidney (*r* = 0.953, *p* = 0.000) as well as for the right kidney (*r* = 0.955, *p* = 0.000), which were also statistically significant. For the left kidney, most of the values of DTPA and MDCT differences were between ± 1.96 s.d. and only few values of these differences were outside ± 1.96 s.d. A similar result was also observed in the right kidney, which is consistent with other previous studies.^3,5,6,9,16^ Summerlin et al.^3^ had reported in their study the mean (±s.d.) renography determined right split function in a sample of 152 was 49.2% ± 4.3% and ranged from 29% to 63%. Comparable to our study, they observed a strong correlation between the MDCT-derived SRF and renal scintigraphy SRF.

Habbous et al.^25^ performed a systemic review and meta-analysis to answer whether CT SRF can replace nuclear SRF in LKD. They reviewed 19 studies and obtained a pooled correlation of *r* = 0.74. Importantly, they found that for every 1% increase in the split renal volume, the SRF increased by 0.76%. A marked difference was noted, however, in the practice of obtaining a diuretic renogram before renal donation, with some centres performing a renogram for all patients, whilst others performing it only when the GFR is below the donation threshold. As a result, they reported a false negative rate of 14% for predicting SRF for renal donation. However, this is because of the selective use of diuretic renogram when the discrepancy is > 1 cm in renal size or > 10% in SRF. If all the candidates were tested, this rate would be much less.

### Limitations

This study has a large sample size unlike most earlier studies and is adequately powered. Being a prospective study from a single institute, the selection criteria and follow-up protocols are well defined, and there is no attrition. The main limitation of this study is that post-nephrectomy outcomes of all donors were not compared with preoperative CT SRF/ GFR, which could have yielded more insights into the role of CT in the prediction of residual renal function after donor nephrectomy.

## Conclusion

Multidetector computed tomography volumetry-attenuation derived estimation of SRF for living renal donors could be an alternative to renal scintigraphy-based SRF estimation. In addition, it makes the evaluation faster, more cost effective with reduced utilisation of imaging resources, and avoids additional radiation and nuclear pharmaceutical hazards.
